# Changes in Momentary Experience Were Associated With the Onset of the COVID Pandemic

**DOI:** 10.1093/geroni/igab046.2086

**Published:** 2021-12-17

**Authors:** Arthur Stone, Hio Mak

**Affiliations:** 1 University of Southern California, University of Southern California/Los Angeles, California, United States; 2 University of Southern California, University Of Southern California, California, United States

## Abstract

We explored the COVID-19 pandemic’s effects on daily experience using momentary recordings of affect, activities, locations, and social interactions, documenting changes in the pandemic’s early stages. 123 individuals 50 years or older from an ongoing panel study completed 1-week bursts of Ecological Momentary Assessment (6/day) in March, May, and July. A pronounced spike in negative affect and decrease in positive affect was observed in late March compared with early March, which in May and June returned to early March levels. Levels of fatigue, however, did not follow this pattern. Being with one’s spouse/significant other and family also increased, then decreased. Working and interacting with others dropped from early to late March and then remained steady; doing chores had the opposite pattern. Regarding location, being at the workplace dropped from early to late March and remained steady, and being at home had the opposite pattern. Additional analyses explored these patterns.

